# Aging Biomarkers for Evaluating the Life Style Quality of Elderly and Senile People

**Published:** 2018-05

**Authors:** Gulnur DOSZHANOVA, Aigul ABDULDAYEVA, Kulsara DOSMAMBETOVA

**Affiliations:** Dept. of Preventive Medicine and Nutrition, JSC “Astana Medical University”, Astana, Kazakhstan

## Dear Editor-in-Chief

The notion “biological”, “physiological” and “psychological” ages are currently used in Gerontology as a biomarker for active aging and the factors affecting the length and quality of elderly people life which are integral indicators of the human health level, reflecting a reserve body potential. Studying these indicators is justified not only because of predicting the degree of extension, but also in determining the physiological and psychological state of the organism as well. It is important the “quantity” and “quality” of the lived years.

The study was conducted in 2015 in Astana, Kazakhstan involved 420 respondents aged 60–89 year. Evaluation of biological age (BA) was conducted by the method of V. Voitenko included anthropometric indices, blood pressure measurement, spirometry, breath-exhale, the statistical balance and subjective assessment of their own health. In case of exceeding the biological age the value of its proper value (BA>PV), then accelerated aging was set, in the opposite case (BA<PV) – it decelerated. Physiological age of elderly people has been studied based on the evaluation of functional reserves of the cardiorespiratory system by the Skibinskaya index and adaptation potential, also based on the determination of the individual level of physical health of the respondents on the index of physical condition (1). International questionnaire SF-36 was used to determine the quality of life (QOL).

The decision to participate in the study was taken by the respondent independently and voluntarily in accordance with the legislation of Kazakhstan and the principles of the Helsinki Declaration. Permission to conduct the study was approved by the local Ethics Committee of JSC “Astana Medical University» №11 from 12 March 2015.

The significant differences have been established (*P*<0.001), depending on the sex of the respondents in all biomarkers of aging as compared to their average rate (Table 1). Certain differences between BA and CA, PA and CA, BA and PV respondents within each group indicated the different levels of their functional state and the rate of aging, which was the prerequisite for the detailed analysis of the aging rate of the respondents. The result of detailed analysis has revealed that BA in 94 respondents outpaced the proper BA in an average of 4.15±0.36 yr, and in 326 respondents was behind it at 14.34 ± 0.27 yr. The difference between biological and proper biological age in women are four times greater than the difference compared with men (*P*<0.001), also exceeds the critical level between normality and pathology (7.69±1.17 yr). In order to form more complete information about the degree of aging of the organism, we evaluated the indices of their physiological age, characterized by insufficient adaptation of the organism to the environment, low reserve capacity of the cardiorespiratory system and the average level of the physical condition of the respondents. Significant differences were found in the comparative analysis result of QOL indicators for assessing people with acceleration and deceleration of aging.

**Table 1:** Some biomarkers detected in evaluating the rate of aging

***Index***		***Both sexes (n=420)***	***Men (n=222)***	***Women (n=198)***
Calendar age (CA)		73.22±0.21	69.92±0.35; p2	72.66±0.4
Psychological age (PA) (years)		56.1±0.24; p*1	57.47±1.67	55.82±1.51
Biological age (BA) (years)		55.38±0.27; p*1	63.93±0.27; p2	45.99±0.5
Roper value (PV) (years)		3.13±0.17; p*1	67.17±0.25; p2	59.26±0.23
BA: PV		0.88±0	0.96±0; p2	0.78±0.01
BA- PV		−7.74±0.25	−3.24±0.32; p2	−13.35±0.56
**The markers of BA:**	**Average norms**			
Body weight (kg)	67.5±1.5	71.59±0.27	74.44±0.56; p2; p3	69.09±0.61
Vital capacity of-the lungs (ml)	3251.00±55.9	1951.11±11.73; p3	2171.11±24.68; p2; p3	1941.11±20.84; p3
Systolic blood pressure (mm Hg)	119.7±1.98	137.26±0.48; p3	138.44±0.78; p2; p3	133.92±1.02; p3
Diastolic blood pressure (mm Hg)	68.74±1.03	87.49±0.25; p3	87.17±0.46; p1;p3	88.88±0.55; p3
Pulse pressure(mm Hg)	42.4±1.3	49.77±0.36; p3	51.28±0.66; p1; p3	45.04±0.82
Respiratory arrest occurs on inspiration (sec.)	41.2±0.7	24.61±0.28; p3	24.99±0.57; p3	24.5±0.64; p3
The duration of static balancing on one leg (sec).	31.3±1.4	12.49±0.54; p3	11.92±0.98; p1;p3	8.83±0.81; p3
Subjective assessment of health points	12.01±0.2	16.54±0.11; p4	14.38±0.23; p3	14.3±0.27; p3

Note:

p*1 - confidence *P*<0.001 of index BA and PA in comparison with CA и PV; p1 - confidence *P*<0.05 of index in the male group in comparison the female; p2 - confidence *P*<0.001 of index in the male group in comparison the female; p3 - confidence *P*<0.001 of index in comparison with the norm; p4 – confidence *P*<0.05 of index in comparison with the norm

The level of life activity (49.4±0.68 and 58.5 ± 1.99 points, *P*<0.001) and general health (48.4 ± 1.13 and 55.8 ± 2.14 points, *P*<0.01) was significantly higher during slow rate of aging. Whereas, role (48.2 ± 2.86 and 34.3 ± 4.68 points, *P*<0.01) and physical functioning (67.7 ± 1.24 and 53.4 ± 3.32 points, *P*<0.001) were less pronounced in slow rate of aging. It was established a nonzero feedback on four indicators of life quality and the biological age when physical performance (r = −0.76), role emotional functioning (r = −0.56), vital activity (r = −0.57) and mental health (r = − 0.57) were significantly reduced, with increasing the biological age of the respondents.

This study demonstrated the possibility of integration of the most technically accessible biomarkers of aging in the groups, less correlated with each other, but sufficiently well correlated within the group, which made it possible to evaluate the quality of life and to describe the profile of aging in terms of cardiovascular, respiratory and neuropsychiatric systems, that particularly important for the formation of qualitative longevity of a specific individual.

Thus, the quality of life in elderly and senile people depends on the characteristics of their adaptation to daily activities, the functionality of individual systems of the organism, the level of physical qualities of the respondents. Taking into consideration uneven rate of changing of aging markers, it is important to pay a special focus on the condition of the health from the young age. All above mentioned prove that the aging rate of men and women in gerontological population groups in Kazakhstan is comparable to the general regularities of changes of lifetime population in the world.
